# Economic modeling in HIV for maraviroc in France in treatment experienced patients. Results from the ARAMIS 2011 model

**DOI:** 10.1186/1742-4690-9-S1-P60

**Published:** 2012-05-25

**Authors:** Nicolas Despiegel, Felicitas Kuehne, Monique Martin, Ahmed Shelbaya

**Affiliations:** 1 Vp Uk French Heor Operations at Optuminsight, Uxbridge, UK

## Introduction

To update an existing and previously published economic (micro-simulation model) in HIV (ARAMIS) to reflect current treatment patterns and to evaluate the cost-effectiveness of maraviroc (MVC) in France.

## Materials and methods

A systematic literature review was carried out in PubMed to identify all articles published in the past 5 years to provide new data to update the existing model. A total of 1964 abstracts were identified on opportunistic infections (OIs), health consequences (effects on health over the longer term e.g. cancer), costs, quality of life, adherence, resistance and efficacy of treatments. In addition, current guidelines were identified and reviewed. New data were included for OIs, costs, treatments and LT health consequences. Treatments focussed on maraviroc, etravirine (ETR) and raltegravir (RAL) including optimised background therapy (OBT) for treatment-experienced patients at the model’s start. All relevant trials with these agents were identified, data were extracted and used in a meta-analysis (results not provided here) which provided relative efficacy data at 48 and 96 weeks. Treatment algorithms were updated based on guidelines and expert opinion. Costs were at the 2011 level based on official sources. There were insufficient data on adherence to include this in the model.

## Results

The updated version of the ARAMIS model indicates that MVC compared to RAL or ETR over a life time is associated with more QALYs (a difference of 0.037 and 0.134 respectively) but higher total costs (a difference of €1,439 and €4,766, respectively). The incremental cost effectiveness ratio for MVC compared to RAL or ETR is €39,300 and €35,700. Assuming a threshold of €50,000 MVC can be considered cost-effective compared to RAL and ETR. Life expectancy with MVC was similar to RAL (0.37 month difference) and higher than ETR (1.58 months difference).

## Conclusion

MVC is a cost-effective treatment option for CCR5 tropic treatment-experienced patients in France.

